# Application of Centrifugal Partition Chromatography for Bioactivity-Guided Purification of Antioxidant-Response-Element-Inducing Constituents from Atractylodis Rhizoma Alba

**DOI:** 10.3390/molecules23092274

**Published:** 2018-09-06

**Authors:** Myeong Il Kim, Ji Hoon Kim, Ahmed Shah Syed, Young-Mi Kim, Kevin Kyungsik Choe, Chul Young Kim

**Affiliations:** 1 College of Pharmacy and Institute of Pharmaceutical Science and Technology, Hanyang University, Ansan, Gyeonggi-do 15588, Korea; auddlf1117@hanmail.net (M.I.K.); gg890718@gmail.com (J.H.K.); shahahmed454@gmial.com (A.S.S); ymikim12@hanyang.ac.kr (Y.-M.K.); kkchoe@hanyang.ac.kr (K.K.C.); 2 Department of Pharmacognosy, Faculty of Pharmacy, University of Sindh, Jamshoro 76080, Pakistan

**Keywords:** Atractylodis Rhizoma Alba, centrifugal partition chromatography, antioxidant response element, polyacetylene, sesquiterpenes

## Abstract

Activity-guided separation of antioxidant response element (ARE)-inducing constituents from the rhizomes of Atractylodis Rhizoma Alba was performed by the combination of centrifugal partition chromatography (CPC) and an ARE luciferase reporter assay. From 3 g of the active *n*-hexane fraction, one polyacetylene, (6*E*,12*E*)-tetradeca-6,12-dien-8,10-diyne-1,3-diyl diacetate (47.3 mg), and two sesquiterpenes, atractylenolide I (40.9 mg), and selina-4(14),7(11)-dien-8-one (6.0 mg) were successfully isolated by CPC with *n*-hexane–ethyl acetate–methanol–water (8:2:8:2, *v*/*v*). The chemical structures of the isolated compounds were determined by ^1^H- and ^13^C-NMR and ESI-MS. Among the isolated compounds, (6*E*,12*E*)-tetradeca-6,12-diene-8,10-diyne-1,3-diol diacetate and selina-4(14),7(11)-dien-8-one increased ARE activity 32.9-fold and 16.6-fold, respectively, without significant cytotoxicity, when 5 µM sulforaphane enhanced ARE activity 27.1-fold. However, atractylenolide I did not increase ARE activity at 100 µM, and showed cytotoxicity at concentrations over 10 µM.

## 1. Introduction

Oxidative stress is involved in various pathological conditions including ageing, cancer, inflammation, and chronic diseases [1]. Reactive oxygen species can be scavenged by the use of antioxidants including vitamins, carotenoids, and polyphenolic compounds [2]. Induction of antioxidant signaling pathway via upregulation of endogenous antioxidant/phase II detoxifying enzymes may be an alternative defensive approach. The activation of nuclear factor erythroid 2-related factor 2 (Nrf2)-antioxidant response element (ARE) pathway leads to the induction of antioxidant/phase II detoxifying enzymes including heme oxygenase 1, NAD(P)H: quinine oxidoreductase 1, and glutathione *S*-transferase. There is growing interest in molecules that activate the Nrf2-ARE signaling pathway [3].

Atractylodis Rhizoma Alba (the rhizome of *Atractylodes macrocephala* Koidz. or *A. japonica* Koidz. ex Kitam.) is a traditional medicine in Korea, Japan, and China. In Korea, the aerial parts of Atractylodis Rhizoma Alba are eaten as vegetables, while the rhizomes are used as medicinal herbs. According to Korean traditional medicine publications, Atractylodis Rhizoma Alba has warm properties and a bitter taste; it strengthens the stomach and spleen, resolves dampness, and stops sweating [4,5].

Previous phytochemical investigations led to the isolation of sesquiterpenes, polyacetylenes and phenolic acids from Atractylodis Rhizoma Alba, which have various biological effects such as antioxidants, gastroprotective and anti-inflammatory activities, as well as inhibiting aromatase and the farnesoid X receptor and modulating progesterone receptors [6,7,8,9,10,11,12].

The separation and purification of the bioactive molecules from natural products requires repeated chromatography, and in some cases the active ingredient does not elute by absorption to the solid phase [13]. To overcome these problems, centrifugal partition chromatography (CPC) is a viable alternative method. CPC is a preparative, support-free, liquid–liquid chromatographic technique, which is an efficient tool for bioactivity-guided isolation of natural ingredients that allows increased sample loading and the absence of sample loss due to irreversible sample absorption to a solid column [14,15,16,17,18].

**Figure 1:**
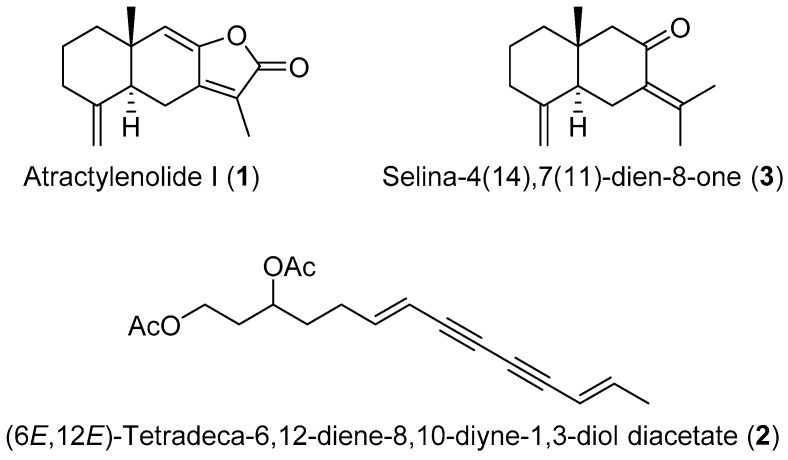
Chemical structures of purified compounds **1**–**3**.

The aim of this study was to develop a simple activity-guided isolation procedure for ARE-inducing compounds from the crude extract of *A. macrocephala* by CPC and an ARE luciferase reporter assay in HepG2 cells. As a result, two sesquiterpenes including atractylenolide I (**1**) and selina-4(14),7(11)-dien-8-one (**3**), and one polyacetylene, (6*E*,12*E*)-tetradeca-6,12-diene-8,10-diyne-1,3-diol diacetate (**2**) (Figure 1) were obtained by one-step CPC. The ARE induction activity and cytotoxicity of these compounds **1**–**3** were also evaluated.

## 2. Results and Discussion

### 2.1. ARE Luciferase Assay and HPLC Analysis of the Crude Extract and Sub-Fractions

**Figure 2:**
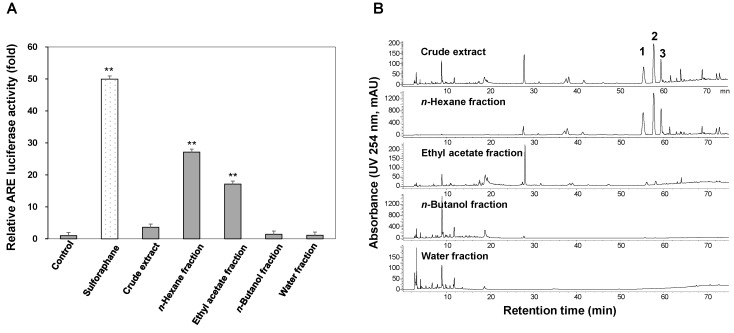
ARE luciferase activities in HepG2 cells (**A**) and HPLC chromatograms of the crude extract and its sub-fractions (**B**). The relative ARE luciferase activities were measured in the lysates of HepG2 cells that were stably transfected with pGL4.37 after exposure to 30 µg/mL of crude extract or sub-fractions for 12 h. Data are presented as means ± S.E. (*n* = 5). ** *p* < 0.01 (compared with the vehicle-treated control).

Based on the ARE luciferase assay, the *n*-hexane fraction was the most active, followed by the ethyl acetate fraction. At a concentration of 30 µg/mL, the *n*-hexane and ethyl acetate fractions enhanced ARE activity 27.1 and 17.1-fold, respectively. Sulforaphane, the positive control, increased ARE activity 49.9-fold at a concentration of 5 µM (Figure 2A). 

HPLC analysis of crude samples revealed that compounds **1**–**3** were major constituents of *A. macrocephala* in the *n*-hexane fraction (Figure 2B). Based on its ARE-enhancing activity and HPLC chromatogram, the *n*-hexane fraction was selected to purify active molecules by CPC.

### 2.2. Optimization of the Two-Phase Solvent System and CPC Operation

A two-phase solvent system with petroleum (60–90 °C)–ethyl acetate–ethanol–water (4:1:4:1, *v*/*v*) has previously been reported for the separation of actractylon and atractylenolide III from *A. macrocephala* by high-speed counter-current chromatography [19]. Based on this result, several two-phase solvent systems consisting of *n*-hexane–ethyl acetate–methanol–water at different ratios were tested to calculate *K*-values for major constituents of the *n*-hexane fraction (Table 1).

CPC was performed with a solvent ratio of 8:2:8:2 (*v*/*v*), although the *K*-value was better at a ratio of 9:1:9:1 (*v*/*v*) for the three major compounds **1**–**3** in the *n*-hexane fraction. The peak at 28.6 min, a major component in ethyl acetate fraction, was also expected to exert ARE-inducing activity (Figure 1). Therefore, this peak is more polar than compounds **1**–**3**, which is why the two-phase solvent system with 8:2:8:2 was more suitable for polar conditions than the 9:1:9:1 system.

The retention time of each compound **1**–**3** in CPC was calculated using a general chromatographic retention equation (V_R_ = V_M_ + K_C_V_S_, V_R_: the retention volume of the solute, V_M_: the mobile phase volume, K_C_: the partition coefficient, V_S_: the stationary volume) [20]. In this experiment, SCPC-1000 was used (column volume: 1000 mL, flow rate: 10 mL/min, mobile phase retention: 54%). Therefore, the calculated retention time for each peak was 102.16 min (peak **A**), 140.24 min (peak **B**), and 251.12 min (peak **C**).

**Figure 3:**
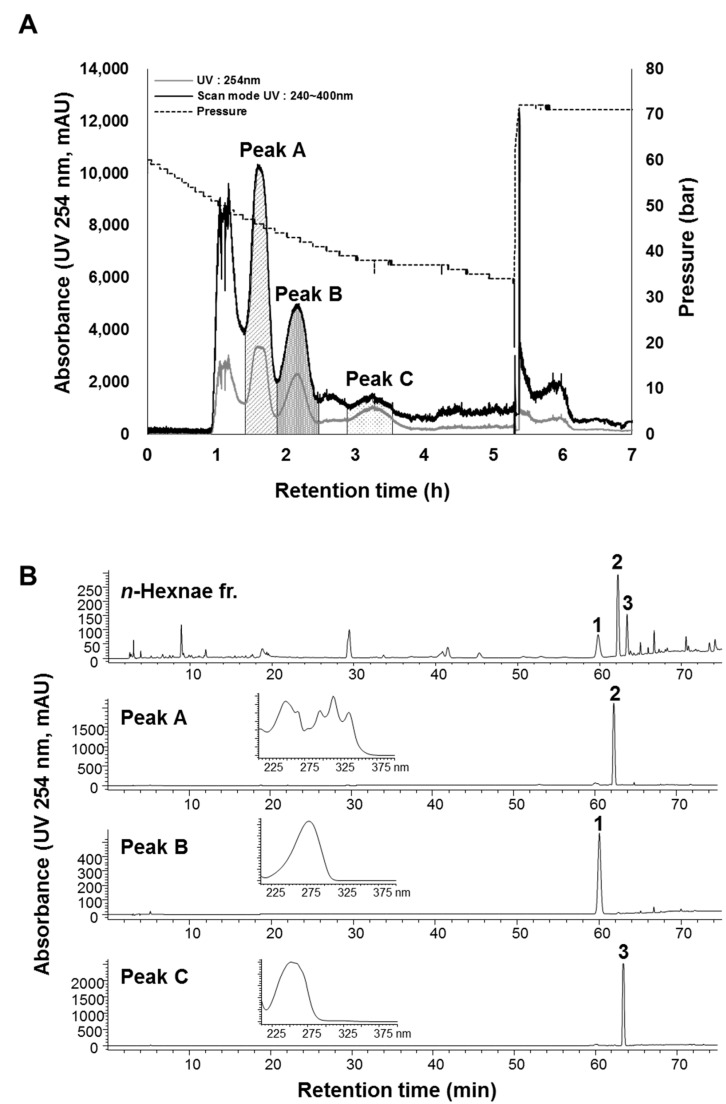
CPC chromatogram of the *n*-hexane fraction (**A**) and HPLC chromatograms and UV spectra of the *n*-hexane fraction and purified peaks (A–C) (**B**). CPC conditions: two-phase solvent system, *n*-hexane–ethyl acetate–methanol–water (8:2:8:2, *v*/*v*), descending mode; mobile phase, lower (more hydrophilic) phase; flow rate, 10 mL/min; rotation speed, 1100 rpm; monitored at 254 nm and scan mode (240–400 nm). Peak **A**: (6*E*,12*E*)-tetradeca-6,12-diene-8,10-diyne-1,3-diol diacetate (**2**), peak **B**: atractylenolide I (**1**) and peak **C**: selina-4(14),7(11)-dien-8-one (**3**).

As shown in Figure 3, the *n*-hexane fraction (3 g) separated excellently into three major response CPC peak fractions: **A** (90–110 min), **B** (120–146 min), and **C** (178–227 min) (Table 2). The first two peaks (**A** and **B**) eluted as expected, but the third peak (**C**) eluted earlier than the calculated time. This difference is due to the gradual outflow of the stationary phase, which can be observed by a reduction in the operating pressure (Figure 3A).

Each CPC peak fraction from **A** to **C** was concentrated to yield 47.3 mg of peak **A**, 40.9 mg of peak **B** and 6.0 mg of peak **C**. Afterward, the fractions obtained from the CPC chromatogram were analyzed via analytical HPLC. Fraction peaks **A**–**C** from the CPC with a descending mode corresponded to the peak order of **2**, **1** and **3** in the HPLC chromatogram in Figure 3. The purities of atractylenolide I (**1**), (6*E*,12*E*)-tetradeca-6,12-dien-8,10-diyne-1,3-diyl diacetate (**2**) and selina-4(14),7(11)-dien-8-one (**3**) were demonstrated to be >99%, 95% and 97%, respectively, according to the area of the HPLC peaks at 254 nm (Figure 3B).

### 2.3. ARE-Activating Effect of Purified Compounds ***1***–***3***

**Figure 4:**
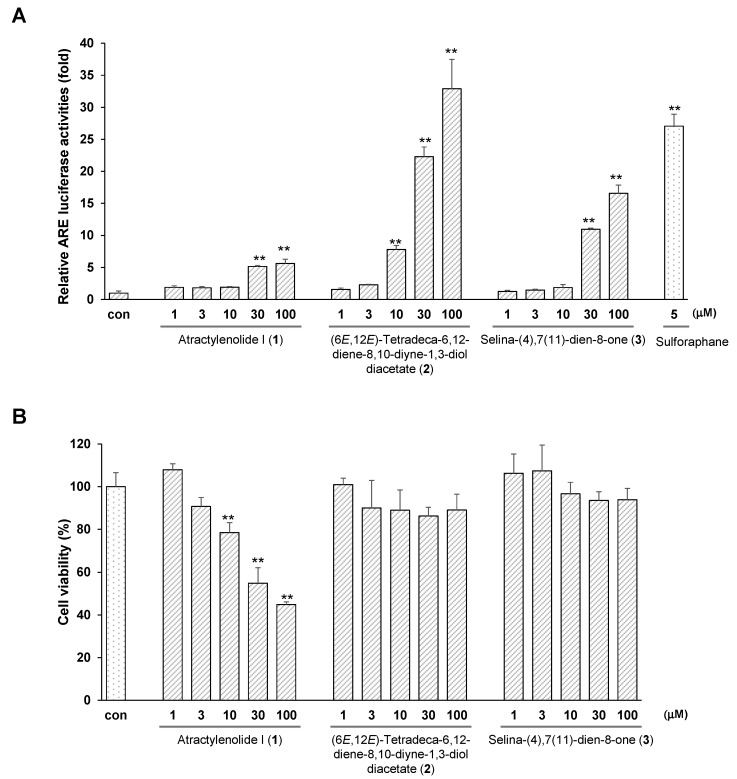
ARE luciferase activities (**A**) and cytotoxicity (**B**) of isolated compounds **1**–**3** in HepG2 cells. The relative ARE luciferase activities were measured in the lysates of HepG2 cells that were stably transfected with pGL4.37 after exposure to 1–100 µM of each compound for 12 h. Cell viability was measured by the MTT assay. Data are presented as means ± S.E. (*n* = 5). **, *p* < 0.01 (compared with the vehicle-treated control).

The capability of the isolated compounds **1**–**3** to augment ARE activity was assessed by a luciferase assay in HepG2 cells. (6*E*,12*E*)-Tetradeca-6,12-diene-8,10-diyne-1,3-diol diacetate (**2**) and selina-4(14),7(11)-dien-8-one (**3**) enhanced ARE activity in a dose-dependent manner (Figure 4A) without significant cytotoxicity. At 100 µM, compounds **2** and **3** increase ARE activity 32.9-fold and 16.6-fold, respectively, without significant cytotoxicity, when 5 µM sulforaphane enhanced ARE activity 27.1-fold. However, atractylenolide I (**1**) did not increase ARE activity at 100 µM, and showed cytotoxicity at concentrations over 10 µM (Figure 4B).

In previous studies, (6*E*,12*E*)-tetradeca-6,12-diene-8,10-diyne-1,3-diol diacetate (**2**) exhibited matrix metalloproteinase-13 downregulating activity in IL-1β-treated SW1353 chondrocytes [21] and anti-methicillin-resistant *Staphylococcus aureus* activity [22], and selina-4(14),7(11)-dien-8-one (**3**) inhibited melanogenesis via reduction of expression of the melanogenic protein that regulated the mRNA level of tyrosinase and tyrosinase-related proteins 1 and -2 [6].

Our results suggest that (6*E*,12*E*)-tetradeca-6,12-diene-8,10-diyne-1,3-diol diacetate (**2**) and selina-4(14),7(11)-dien-8-one (**3**) might reduce oxidative stress by enhancing cellular antioxidant systems without cytotoxicity and compounds **2** and **3** were major active molecules for ARE induction activity in *n*-hexane extract of *A. macrocephala*.

## 3. Materials and Methods

### 3.1. Apparatus and Materials

CPC was performed on an Armen fully integrated SCPC-100+1000 CPC spot instrument (Armen Instruments, St-Ave, France). This instrument is a fully automated system consisting of a CPC column compartment (1000 mL rotor made of 21 stacked disks with a total of 1512 twin cells), a pump, an injector, a UV/vis detector, a fraction collector, a digital screen flat PC, and Armen Glider CPC software. The HPLC analysis was performed by an Agilent 1260 HPLC system (Agilent Technologies, Palo Alto, CA, USA): G1312C binary pump, a G1329B autosampler, a G1315D DAD detector, a G1316A column oven and ChemStation software. HPLC-grade solvents were purchased from Fisher Scientific (Pittsburgh, PA, USA). All other organic solvents used for extraction and CPC operation were analytical grade and obtained from Daejung (Gyonggi-do, Korea). 

### 3.2. Plant Material and Preparation of Crude Extracts

The dried rhizomes of *A. macrocephala* were purchased from the Kyungdong oriental herbal market, Seoul, Korea, in October 2015, and identified by one of author (Dr. CY Kim). A voucher specimen was deposited in the Herbarium of the College of Pharmacy, Hanyang University (HYUP-AM-001). The dried rhizomes (309 g) were ground and extracted in 4 L methanol three times for 3 h under reflux. The methanol solution was evaporated by rotary evaporator to obtain 46 g of extract. The extract was suspended in water and successively fractionated by *n*-hexane, ethyl acetate, and *n*-butanol. The fractions obtained were 9.78 g of *n*-hexane, 6.08 g of ethyl acetate, and 2.92 g of *n*-butanol.

### 3.3. Selection of the Two-Phase Solvent System

The two-phase solvent system was selected according to the *K*-values of the target compounds as a series of solvent systems of *n*-hexane–ethyl acetate–methanol–water. Briefly, approximately 2 mg of the sample was added to each test tube, 2 mL of each phase of a pre-equilibrated two-phase solvent system was added, and the contents were thoroughly mixed. After equilibration, 10 µL of the upper and lower phases were analyzed by HPLC at 254 nm. The *K* value was calculated as the peak area of each compound in the upper stationary phase divided by that of the lower mobile phase.

### 3.4. CPC Separation Procedure

According to the partition coefficient, *n*-hexane–ethyl acetate–methanol–water (8:2:8:2, *v*/*v*) was used as the two-phase solvent system for CPC separation. The two-phase system was prepared in a 4000-mL separation funnel by adding 1200 mL of *n*-hexane, 300 mL of ethyl acetate, 1200 mL of methanol, and 300 mL of water (volume ratio 8:2:8:2). The separation funnel was shaken vigorously and the solvent mixture was thoroughly equilibrated at room temperature. The upper and lower phases were separated and degassed by sonication for 1 h before use.

First, the rotor was filled with the stationary phase (upper phase), and then the rotor was set at 1100 rpm and the lower, more hydrophilic phase was pumped into the channel at a flow rate of 10 mL/min in descending mode. When the stationary phase and mobile phase in the rotor were equilibrated (stationary phase retention: 54%), 3 g of the *n*-hexane fraction dissolved in the 10-mL mixture of stationary phase and mobile phase (1:1, *v*/*v*) was injected. The effluent was monitored at 254 nm and in scan mode (240–400 nm). Each peak fraction was collected according to the chromatogram to obtain three CPC peak fractions **A**–**C**. The fractions were concentrated and analyzed by HPLC.

### 3.5. HPLC Analysis and Identification of the CPC Peak Fractions

The crude extract and each peak fraction obtained by CPC were analyzed by HPLC with an INNO C18 analytical column (4.6 × 250 mm, 5 μm). The mobile phase was composed of acetonitrile containing 0.1% formic acid (A) and water containing 0.1% formic acid (B). The gradient elution conditions were as follows: initial 0 min A:B (10:90, *v*/*v*), 14 min A:B (25:75), 15 min A:B (43:57), 25 min A:B (43:57), 29 min A:B (51:49), 55 min A:B (51:49), 65 min A:B (100:0) and 75 min A:B (100:0). The column temperature was 40 °C, the mobile phase flow rate was 1.0 mL/min, and the detection wavelength was 254 nm. The injection volume was 10 µL.

### 3.6. Structural Identification

Identification of the CPC purified compounds was performed by ESI-MS with an Advion compact mass (Advion, Ithaca, NY, USA) and NMR. The ESI-MS spectra conditions were as follows: positive ion mode; mass range, *m*/*z* 100–1200; capillary temperature, 200 °C; capillary voltage, 150 V; source voltage offset, 30; source voltage span, 10; source gas temperature, 150 °C; and ESI voltage, 3500 V. ^1^H-NMR (400 MHz) and ^13^C-NMR (100 MHz) were measured on a Bruker model digital Avance III 400 NMR in CDCl_3_. The NMR spectra were processed by the MestReNova 9.0 software (Mestrelab Research, Santiago de Compostela, Spain).

### 3.7. Structural Identification of the Purified Compounds

The structures of **1**–**3** were identified as atractylenolide I (**1**), (6*E*,12*E*)-tetradeca-6,12-diene-8,10-diyne-1,3-diol diacetate (**2**) and selina-4(14),7(11)-dien-8-one (**3**) by ^1^H- and ^13^C-NMR data compared with previously published data [6,7,8,10].

### 3.8. Cell Culture and Cell Viability

Human hepatoma (HepG2) cells were obtained from American Type Culture Collection (ATCC, Manassas, VA, USA) and cultured in a DMEM medium supplemented with 10% FBS and 1% penicillin–streptomycin in a humidified atmosphere containing 5% CO_2_ in air at 37 °C. For determination of cell viability, 4 × 10^4^ cells/well were seeded in 96-well plates and incubated overnight. Cells were treated for 12 h with various concentrations of the isolated compounds **1**–**3**. Samples were dissolved in DMSO and the final DMSO concentration did not exceed 0.1%, which did not affect cell cytotoxicity. Cells were then exposed to 0.5 mg/mL MTT for an additional 1 h at 37 °C. The media was removed and 100 μL of dimethylsulfoxide was added to dissolve the formazan crystals produced in each well. Absorbance was measured at 570 nm using an Infinite M200 PRO microplate reader (Tecan, Salzburg, Austria).

### 3.9. ARE Luciferase Reporter Assay

Construction of the HepG2-ARE cells (transfected Pgl4.37 [luc2P/ARE/ Hygro] (Promega)) was reported previously [23]. HepG2-ARE cells were cultured in DMEM high glucose media supplemented with 10% FBS, 1% penicillinstreptomycin and 1% hygromycin B. HepG2-ARE cells were plated at a density of 1 × 10^5^ cells/well in 24-well plates and allowed to reach 70–80% confluence. After 12 h of serum starvation, cells were incubated with the extract, sub-fractions or isolated compounds **1**–**3** for 12 h. After treatment, cells were washed twice with ice-cold phosphate-buffered saline, and 120 μL of passive lysis buffer (Promega, Madison, WI, USA) was added to each well and reacted at 4 °C for 30 min. The supernatant was transferred to an e-tube and centrifuged at 13,000 rpm for 15 min at 4 °C, and 60 µL of the supernatant was incubated with 30 µL of the luciferase assay substrate (Promega, Madison, WI, USA) in a 96-well plate. Luminescence was measured by an EnSpire multimode plate reader (PerkinElmer, Waltham, MA, USA). Sulforaphane (5 μM) (Calbiochem, Darmstadt, Germany) was used as a positive control.

### 3.10. Statistical Analysis

All data are reported as means ± S.E. The statistical significance of differences between treatments was assessed using the Student’s *t*-test. Probability values less than 0.01 were considered significant.

## 4. Conclusions

In this study, bioactivity-guided isolation for ARE-inducing compounds was developed using a combination of CPC and an ARE luciferase reporter assay. One-step CPC enabled the isolation of two sesquiterpenes including atractylenolide I (**1**) and selina-4(14),7(11)-dien-8-one (**3**) and one polyacetylene, (6*E*,12*E*)-tetradeca-6,12-diene-8,10-diyne-1,3-diol diacetate (**2**). Among these compounds, compounds **2** and **3** exerted ARE-inducing activity. The overall results of this study demonstrated that CPC is an efficient tool for bioactivity-guided purification from natural products.

## Figures and Tables

**Table 1:** Partition coefficients (*K*) of *n*-hexane fraction of *A. macrocephala.*

Solvent Systems (HEMW, *v*/*v*)	*K*-Value		
	Atractylenolide I (1)	(6*E*,12*E*)-Tetradeca-6,12-dien-8,10-diyne-1,3-diyl diacetate (2)	Selina-4(14),7(11)-dien-8-one (3)
9:1:9:1	0.82	0.40	1.93
8:2:8:2	1.54	0.86	3.62
7:3:7:3	3.37	2.35	7.80

The *K*-value is defined as the peak area of the compounds in upper stationary phase divided by the peak area of those in the lower mobile phase. H: *n*-hexane, E: ethyl acetate, M: methanol, W: water.

**Table 2:** Comparison of CPC retention time calculated by *K*-values and operation result in the two-phase solvent system of *n*-hexane–ethyl acetate–methanol–water (8:2:8:2, *v*/*v*).

CPC Peaks	Peak A	Peak B	Peak C
Compounds **1**–**3**	(6*E*,12*E*)-tetradeca-6,12-dien-8,10-diyne-1,3-diyl diacetate (**2**)	atractylenolide I (**1**)	selina-4(14),7(11)-dien-8-one (**3**)
*K*-values of compounds **1**–**3**	0.86	1.54	3.62
Retention time (calculated)	102.16 min	140.24 min	251.12 min
Retention time (experimental data)	90–10 min	120–146 min	178–227 min

CPC operating conditions: CPC volume, 1000 mL; flow rate, 10 mL/min; mobile phase retention, 54%.

## Supplementary Materials

Supplementary File 1
