# Supplementary material for: Application of Centrifugal Partition Chromatography for Bioactivity-Guided Purification of Antioxidant-Response-Element-Inducing Constituents from Atractylodis Rhizoma Alba
Source: Molecules. 2018 Sep 6;23(9):2274. doi: 10.3390/molecules23092274 (PMC6225303; doi:10.3390/molecules23092274)
Supplement: Supplementary File 1 [file molecules-23-02274-s001.pdf]

Application of Centrifugal Partition Chromatography for Bioactivity-Guided Purification of Antioxidant-Response-Element-Inducing Constituents from Atractylodis Rhizoma Alba

Myeong Il Kim [1,†], Ji Hoon Kim [1,†], Ahmed Shah Syed [1,2], Young-Mi Kim [1], Kevin Kyungsik Choe [1] and Chul Young Kim [1,]

ESI-Mass and NMR data of compounds **1–3**.

Atractylenolide I (**1**): ESI-MS (*m/z*), 253 [M + Na]$^+$; ^{1}H-NMR ($CDCl_3$, 400 MHz) δ 5.61 (1H, s, H-9), 4.91 (1H, d, *J* = 1.6 Hz, H-15b), 4.62 (1H, d, *J* = 1.6 Hz, H-15a), 2.69 (1H, dd, *J* = 16.8, 3.9, H-6b), 2.52 (1H, ddd, *J* = 16.8, 13.2, 1.2, H-6a), 2.36 (1H, m, H-5), 2.36 (1H, m, H-3b), 2.06 (1H, m, H-3a), 1.90 (3H, d, *J* = 1.8, H-13), 1.69 (2H, m, H-2), 1.61 (2H, m, H-1), 0.94 (3H, s, H-14); ^{13}C-NMR ($CDCl_3$, 100 MHz) δ 171.5 (C-12), 148.5 (C-4), 148.3 (C-7), 148.1 (C-8), 120.6 (C-11), 119.3 (C-9), 107.6 (C-15), 48.5 (C-5), 39.2 (C-1), 38.2 (C-10), 36.3 (C-3), 23.1 (C-6), 22.8 (C-2), 18.7 (C-14), 8.6 (C-13).

(6*E*,12*E*)-Tetradeca-6,12-diene-8,10-diyne-1,3-diol diacetate (**2**): ESI-MS (*m/z*), 325 [M + Na]$^+$; ^{1}H NMR ($CDCl_3$, 400 MHz) δ 6.32 (1H, m, H-6), 5.58 (1H, s, H-12), 5.54 (1H, s, H-7), 4.99 (1H, m, H-3), 4.08 (2H, t, *J* = 6.4 Hz, H-1), 2.17 (2H, dd, *J* = 14.6 Hz, 7.4 Hz, H-5), 2.05 (3H, s, H-3-OAc), 2.04 (3H, s, H-13-OAc), 1.87 (2H, dt, *J* = 6.4 Hz, 2.6 Hz, H-2), 1.82 (3H, *J* = 6.8 Hz, 1.6 H, H-14), 1.68 (2H, m, H-4); ^{13}C-NMR ($CDCl_3$, 100 MHz) δ 171.1 (-O-**C**=O), 170.8 (-O-**C**=O), 146.7 (C-6), 143.7 (C-13), 110.0 (C-12), 109.6 (C-7), 80.2 (C-11), 79.4 (C-8), 73.2 (C-10), 72.4 (C-9), 70.5 (C-3), 60.8 (C-1), 33.2 (C-2), 33.1 (C-4), 29.3 (C-5), 21.2 (CH3), 21.1 (CH3), 19.1 (C-14).

Selina-4(14),7(11)-dien-8-one (**3**): ESI-MS (*m/z*), 241 [M + Na]$^+$; ^{1}H-NMR ($CDCl_3$, 400 MHz) δ 4.86 (1H, brs, H-15a), 4.61 (1H, brs, H-15b), 2.73 (1H, d, *J* = 10.9 Hz, H-6a), 2.37 (1H, m, H-3a), 2.29 (2H, s, H-9), 2.26 (1H, m, H-6b), 2.23 (1H, m, H-5), 2.04 (1H, m, H-3b), 1.99 (3H, s, H-13), 1.82 (3H, s, H-12), 1.64 (1H, m, H-2a), 1.53 (1H, m, H-1a), 1.52 (1H, m, H-2b), 1.42 (1H, dd, *J* = 16.6, 4.4, H-1b), 0.77 (3H, s, H-14); ^{13}C-NMR ($CDCl_3$, 100 MHz) δ 203.9 (C-8), 149.2 (C-4), 142.7 (C-11), 131.7 (C-7), 107.1 (C-15), 57.8 (C-9), 47.2 (C-5), 41.4 (C-1), 38.4 (C-10), 36.9 (C-3), 29.3 (C-6), 23.4 (C-13), 23.3 (C-2), 22.4 (C-12), 17.5 (C-14).
